# Differences in help-seeking tendency in intimate partner violence between Jewish and Arab women in Israel

**DOI:** 10.3389/fsoc.2023.1229924

**Published:** 2023-08-09

**Authors:** Vered Ne'eman-Haviv, Yoel Shafran

**Affiliations:** ^1^Department of Criminology, Ariel University, Ariel, Israel; ^2^Michal Sela Forum, Mevaseret Zion, Israel

**Keywords:** help seeking tendency, intimate partner violence, cultural differences, victimization, support source, mental health

## Abstract

**Objective:**

Intimate partner violence (IPV) has many consequences for the physical and mental health of the victims. One strategy for coping with IPV is to turn to formal and informal sources for help. The purpose of the present study was to examine the differences in help seeking tendency in cases of IPV between Jewish and Arab women in Israel and the connection to their mental health.

**Method:**

We administered a structured quantitative questionnaire to a sample of 357 Jewish (44.8%) and 439 Arab (55.2%) women.

**Results:**

The findings indicate that Jewish women tend to seek help more often than do Arab women, and that there are differences in the targets they approach. Jewish women turn more frequently to unofficial sources, such as friends, relatives, and associations, whereas Arab women approach more often official sources such as social workers and clergy.

**Conclusions:**

We propose an explanation for the differences based on socio-cultural factors. This study illustrates that it is necessary to act with cultural sensitivity and adapt the help options offered to the culture to which the women belong. This adjustment may encourage more women to apply for support to escape the world of violence.

## Introduction

Intimate partner violence (IPV) refers to behavior that occurs in a current or previous intimate relationship, which causes physical, psychological, or sexual harm. It includes acts of physical aggression, sexual coercion, and various forms of psychological or emotional abuse (Potter et al., 2021). The latter may include humiliating, threatening, and coercive behaviors designed to control the victim and limit her autonomy (Stark and Hester, 2019). Exposure to IPV is a cause of injuries and physical harm, and it contributes to mental health problems including depression, post-traumatic stress disorder (PTSD), and suicidality (Spencer et al., 2019).

Many studies, that examined IPV in different societies and cultures found that it is present regardless of social class, religion, sector, country, or continent, and it exists for many psychological and sociological reasons (Gilbar et al., 2022). In the present study we argue that help-seeking in the wake of IPV, like IPV itself, is the product not only of individual psychological aspects but also of social structure, which includes clear role definitions, norms, and stereotypes. The study focuses on the differences in the tendency to ask for help in cases of IPV between Jewish and Arab women living in Israel, with the understanding that help-seeking depends, among others, on cultural and social codes.

## Literature review

### Sectoral differences in IPV against women in Israel

Israeli society consists of many sectors and population groups, but the phenomenon of IPV is present in all of them (Shwartz et al., 2022). Studies that examined the differences between the diverse groups have found that Arab women born in Israel have the highest rate of IPV victimization, 21%, in the Israeli population (Ben-Porat et al., 2021). In a survey conducted among women of childbearing age in Israel, considerable differences were found in the prevalence of IPV among Arab women born in Israel, Jewish women born in Israel, and immigrants to Israel (67, 27, and 30%, respectively). All types of IPV were more frequent among Arab women and recurrence was higher than the other two groups (Daoud et al., 2020).

Arabs are a minority in Israel. The Arab population of Israel, which includes Muslims, Bedouins, Christians, and Druze, constitutes about 21% of the total population of the country (Central Bureau of Statistics, 2020). The Arab society is different in many respects from Jewish Israeli society. Although the differences are not completely dichotomous and unequivocal, in general, the Arab society is more traditional, collectivist, and patriarchal, and the Jewish society is more individualistic and liberal (Benita, 2017). Despite the modernization processes that Arab society is undergoing, and the increase in the rate of Arab women working outside the home and acquiring higher education, Arab society remains patriarchal, which places women in a low position in the family hierarchy. The woman's roles as a wife and mother, and expectations of her, remain the same even if she carries the additional burden of working outside the home (Ne'eman-Haviv, 2021a). According to Sweet (2019), romantic relationships are the arena where traditional gender ideologies are upheld most strongly. This is also reflected in the phenomenon of violence and murder for the sake of family honor in Arab society in Israel (Ne'eman-Haviv, 2021b).

### Tendency to seek help

IPV has many implications, both physical and mental-emotional (Lo., 2023). Women who are at risk from their partner are likely to experience increased levels of stress, tension, and anxiety, and are required to develop strategies for coping with their physical, mental, and emotional state. At times, they are required to defend their lives (MacGregor et al., 2021). Coping is a regulatory process used to reduce the negative emotional effects of stressful events (van Berkel, 2009). Coping strategies refer to methods adopted and practiced by people as means of dealing with various stressors. Multiple coping strategies are influenced by intrapersonal and environmental factors. One of them is seeking external support and help. The present study examined the probability of seeking external support by Jewish and Arab women, as well as the possible sources of support.

Several articles have proposed a theoretical framework explaining the inclination to seek help in cases of IPV. Lelaurain et al. (2017) conducted a systematic literature review and identified three phases in IPV help-seeking: (1) problem identification; (2) decision to seek assistance; and (3) determining whom to engage for urgent aid.

However, these processes do not occur in isolation but within a social-cultural framework. Liang et al. (2005) assert that the definition of IPV and a person's recognition of being a victim of IPV are influenced by the societal and cultural norms that shape the interaction between gender, class, and culture, as suggested by Waller et al. (2023) in ‘The Theory of Help-Seeking Behavior.' This theory takes into account survivors' sociocultural context, intersectionality, and beliefs, examining how these factors influence the nature and extent of their help-seeking behavior. It postulates that women's beliefs and experiences with available support, as well as their individual agency, impact how and when they seek crisis aid. The Theory of Help-Seeking Behavior includes three key constructs: (1) social context, which explains how survivors' sociocultural context influences their efforts to seek help; (2) beliefs, particularly their lived experiences and reflections on available services and support; and (3) agency, encompassing the strength or power they employ to secure assistance.

Hence, the cultural and social environment is closely linked to the decision to seek help, the barriers that hinder it, and the factors survivors choose to turn to (Padilla-Medina et al., 2023). The barriers encountered during help-seeking depend on survivors' sociocultural context and whether they seek aid from formal providers. These barriers range from shame and stigma to denial of the abuse occurring and aligning with the perpetrator (Waller et al., 2023).

According to this theoretical concept, several studies have shown that the decision to seek help and the decision to whom to turn are affected by social-cultural perceptions (Elias et al., 2019; Lo, 2023; Padilla-Medina et al., 2023; Selestine et al., 2023). Studies conducted in collectivist and patriarchal societies found that women are expected to retain their honor by remaining hidden and maintaining a low profile, restricting their autonomy, and abiding by various constraints to uphold their reputation and that of their family (Aboulhassan and Brumley, 2019).

Hulley et al. (2023) conducted a qualitative meta-synthesis of 47 papers, analyzing samples of women from diverse cultures, including African, Hispanic/Latina, and Asian backgrounds. The study revealed that women in traditional societies often carry symbolic representations of purity within their respective cultures. However, when they deviate from the established cultural norms, they may face consequences such as ostracization, restrictions, or abuse, all of which can destabilize family dynamics. As a result, these women feel immense pressure to adhere to societal expectations and may avoid seeking help from external sources in cases of intimate partner violence (IPV) to protect family stability and avoid further threats.

A study that examined help-seeking by young Arab women in Israel found that they face three categories of barriers: intra- and interpersonal, sociocultural, and sociopolitical (Elias et al., 2019). Beyond the cultural reasons that prevent Arab women from seeking help, other barriers arise from belonging to a minority group in Israel and the existing political conflict between Israel and the Palestinians. Some of these have to do with a lack of trust in the Israeli authorities and with cultural and language gaps. These sociopolitical difficulties may affect both the women's willingness to ask for help and the sources to whom they can turn.

The ways in which women choose the source of support and coping may change and vary between turning to official state agencies (counseling, welfare, legal services) and agents of unofficial support like family and friends. According to Selestine et al. (2023) for women who do decide to seek help, selecting an appropriate source of support brings more challenges. A study that examined help-seeking on a sample of 6,589 American women who experienced domestic violence, and compared women who were part of a social minority with those of a higher social status, revealed that women who were in a social minority sought less help from state aid agencies (Cheng and Lo, 2015). Another study examined the willingness to turn to informal sources of help (family and friends) compared to official sources on a sample of 152 Indian women belonging to a social minority, and found that most of them would prefer to turn to informal sources of assistance (Kim and Hogge, 2015). Additional findings revealed that although women approach informal sources of assistance, they do so with relatively low frequency because they fear that their status in the family will be harmed (Frías and Carolina Agoff, 2015). By contrast, women who are not part of a minority, are more likely to seek assistance in general, mainly from official aid agencies, because they do not encounter the barriers faced by minority groups (Tengku Hassan et al., 2015).

Hence, Arab women in Israel are trapped. On one hand, they are afraid to turn to unofficial sources of assistance within Arab society because they fear damaging the status of the family in the community. On the other hand, turning to formal authorities in Israel is problematic because of the political complexity caused by belonging to a minority group.

The ability to seek help and gain access to sources of support is of great importance (Robinson et al., 2021). It is essential for mental health, protecting a person from the effects of stress (Turner and Brown, 2010). In the case of IPV, the protection is not only for the person's mental state, but often also for her life.

### Research objective

This study examined the willingness of Jewish and Arab women in Israel to ask for help in case of IPV. The cultural differences between the sectors and the differences arising from being part of majority vs. minority groups may affect their tendency to ask for help as well as the sources they choose to approach. Understanding these factors may help devise accurate and culturally appropriate interventions for each group, taking into account and lowering the barriers that prevent seeking help.

### Hypotheses

1. Jewish women will show a greater tendency to seek help than Arab women. Moreover, Jewish women are more likely to seek help from unofficial sources (family and friends), whereas Arab women are more likely to approach official sources that identify with their community (such as Arab-identified social workers).
2. A positive correlation exists between the tendency to seek help and mental wellbeing.
3. A negative correlation exists between the tendency to seek help and demographic variables (age and level of religiosity).

## Methods

### Participants

Seven hundred and ninety-six Israeli women participated in the study, including 357 Jewish (44.8%) and 439 Arab (55.2%) women. Participants' ages ranged 18-75 years (M = 27.33, SD = 9.16) years; 495 (62.1%) of the participants were single, 278 (34.9%) married, and 23 (3%) widowed or divorced; 41.4% defined themselves as religious, 31.8% as traditional, and 23.2% as secular.

An a priori power analysis was conducted using G^*^Power version 3.1.9.7 (Faul et al., 2007) to determine the minimum sample size required to test the study hypothesis. Results indicated the required sample size to achieve 90% power for detecting a medium effect, at a significance criterion of α = 0.05, was *N* = 191 for each group for independent t-test. Thus, the obtained sample size of *N* = 796 is adequate to test the study hypothesis.

### Tools

The study was based on a quantitative questionnaire consisting of three parts:

1. *Demographic questionnaire*, including questions about the participants' age, religion, level of religiosity, and marital status.
2. *Willingness to seek help questionnaire*, which asked participants: “If you were a victim of domestic violence, whom would you be willing to tell about it?” Participants were presented with a list of 11 options and were asked to mark on a scale ranging from 1 (not at all) to 5 (to a very large extent) the likelihood that they would turn to each of them for help. The list of options is shown in Table 1. Cronbach's alpha for the questionnaire was 0.82.
3. *Mental Health Continuum Short Form* (MHC-SF) (Lamers et al., 2011; Shrira et al., 2016). The questionnaire consists of 14 items, each one containing a statement describing mental wellbeing. Participants were asked to rate the frequency with which they experienced each item in the preceding month, on a scale ranging from 1 (not at all) to 6 (every day). Examples of statements: “I felt that my life had meaning and purpose,” “I felt I had a warm and trusting relationship with others.” Cronbach's alpha for the questionnaire was 0.92.

**Table 1:** The probability of seeking help from various sources, by ethnicity.

	**Jewish women (*****N*** = **357)**			**Arab women (*****N*** = **428)**			**T (df)**
	**Low probability**	**Medium probability**	**High probability**	**Low probability**	**Medium probability**	**High probability**	
Father	54.9%	15.4%	29.7%	56.3%	14.5%	29.2%	0.485 (783)
Mother	71.4%	12.9%	15.7%	77%	9.8%	13.3%	1.108 (785)
Brother/sister	64.7%	15.7%	19.6%	71.1%	14.4%	14.6%	1.064 (787)
Other relatives	46.5%	29.4%	24.1%	72.8%	14.3%	12.9%	7.426^*^ (745)
Friend	21.3%	16%	62.7%	48.6%	19.1%	32.3%	11.691^*^ (783)
Teacher	59.1%	21%	19.9%	57.1%	18.5%	24.4%	0.472 (782)
Social worker	40.3%	22.4%	37.3%	39.2%	19.1%	41.7%	0.391 (784)
Physician/nurse	40.6%	22.4%	37%	51.7%	19.6%	28.9%	3.743^*^ (766)
Religious leader	71.1%	11.5%	17.4%	56.3%	15.9%	27.8%	4.317^*^ (783)
Social organization	26.1%	19.6%	54.3%	50%	13.5%	36.5%	7.336^*^ (782)

^*^p < 0.001.

### Research procedure

The study was approved by the University Ethics Committee. The research questionnaires were administered to a convenience sample of Jewish and Arab women in Israel in the course of 2022. Although the sample was not a representative one, it includes representation of participants from various areas in Israel. Emphasis was placed on including participants from Arab, Jewish, and mixed localities (where Jews and Arabs live together). The questionnaires were distributed in Hebrew and Arabic by research assistants from both communities through social networks.

Participants were guaranteed that the survey was anonymous, and that it was intended for research purposes only. The participation in the research was voluntary. We clarified to participants that there were no correct answers to questions and participants were asked only to express their opinions.

### Findings

#### Help-seeking in case of IPV

Confirming our first hypothesis, t-tests for independent samples revealed that there were significant differences between groups in the probability of seeking help for IPV, and the probability of Jewish women seeking help (m = 3.01, df = 0.811) was significantly higher than that of Arab women (m = 2.76, df = 0.795) doing so (t = 4.171, df = 763, *p* < 0.001).

Table 1 presents the likelihood of seeking help from various sources by Jewish and Arab women in Israel.

As shown in Table 1, the rate of help-seeking by both Jewish and Arab women from first-degree family members was not high. About 30% would turn to their fathers with high probability, and less than 20% would turn to their mothers or siblings for help. But there were significant differences between the groups. Most Jewish women (62.7%) indicated that they would turn to a friend, whereas only 32.3% of Arab women did so. Jewish women also indicated greater willingness to seek help from non-first-degree relatives than Arab women did (24.1 vs. 12.9%), physicians or nurses (37.3 vs. 28.9%), and social organizations (54.3 vs. 36.5%). By contrast, Arab women indicated greater willingness than did Jewish women to seek help from social workers (41.7 vs. 37.3%) and of religious leaders (27.8 vs. 17.4%).

#### Correlations between variables

Tables 2, 3, concerning hypotheses 2 and 3, present statistics of the study variables and the correlations between them. We examined the correlations between likelihood to seek help, mental wellbeing, and age using Pearson's test, and the correlations with the level of religiosity with Spearman's correlation.

**Table 2:** Statistics of research variables.

		**Probability of seeking help**	**Mental wellbeing**	**Age**	**Level of religiosity**
**Complete sample**	*N*	764	795	793	795
	Mean	2.88	3.13	27.34	2.18
	SD	0.81	0.78	9.15	0.78
	Min	1	1	18	1
	Max	5	471	75	3
**Jewish women**	*N*	357	357	357	357
	Mean	3.01	3.17	25.75	2.02
	SD	0.81	0.73	8.05	0.87
	Min	1	1.14	19	1
	Max	5	4.71	65	3
**Arab women**	*N*	407	438	436	438
	Mean	2.77	3.09	28.65	2.31
	SD	0.79	0.82	9.78	0.68
	Min	1	1	18	1
	Max	5	4.71	75	3

**Table 3:** Correlations between research variables.

		**Probability of seeking help**	**Mental wellbeing**	**Age**
**Jewish women**	Mental wellbeing	0.272^**^		
	Age	0.182^**^	0.153^**^	
	Level of religiosity	0.098	0.066	−0.191^**^
**Arab women**	Mental wellbeing	0.120^*^		
	Age	−0.175^**^	0.143^**^	
	Level of religiosity	−0.157^**^	0.147^**^	0.125^*^

^*^p < 0.05, ^**^p < 0.001.

As shown in Tables 2, 3, and confirming hypothesis 3, the probability of seeking help correlated significantly with the sense of mental wellbeing. The correlation was stronger in Jewish than in Arab women.

We found a significant correlation between the probability of seeking help in the case of IPV and the participants' age, with opposite trends between Jewish and Arab women. For Arab women, we found a negative correlation, in accordance with hypothesis 4, whereas for Jewish women, we found a positive correlation. Thus, the younger Arab women were, the greater the probability was of their seeking help, whereas for Jewish women, the probability of seeking help was higher for older women.

This finding may be explained by the level of religiosity of the participants. Contrary to hypothesis 4, for Jewish women we found no correlation between the probability of seeking help and level of religiosity. By contrast, for Arab women, we found a negative correlation between these variables. The significant correlation between age and level of religiosity of Arab women explains why older women were less likely to seek help. For Jewish women, the correlation between age and the level of religiosity was negative, therefore we found no correlation between the level of religiosity and the tendency to seek help.

## Discussion

IPV has many consequences for the physical and mental health of the victims (Spencer et al., 2019; Lo, 2023). One of the victims' strategies for coping with IPV is turning to formal and informal sources for help (van Berkel, 2009; Selestine et al., 2023). The present study examined the tendency of Jewish and Arab women in Israel to seek help in case of IPV. The findings revealed a significant connection between the willingness to ask for help and the women's mental health. The mere knowledge that the women have someone to turn to in case of violence appears to lead to better mental wellbeing. An understanding of the barriers that women face in seeking help and of the entities they can approach for help can guide service providers in developing solutions that are culturally appropriate for each community, in accordance with its cultural codes.

The findings of the study reveal that, in general, Jewish women's willingness to seek help is significantly higher than that of Arab women. This gap originates in the differences between the two cultures. Although Jewish society contains, among other, conservative groups, and the differences between the Jewish and the Arab societies do not constitute an extreme dichotomy, the Jewish society is considered to be more liberal, Western, and egalitarian than the Arab society. Arab society in Israel, despite the change it is undergoing, remains traditional, collectivist, and patriarchal (Daoud et al., 2020). Arab culture expects women to be submissive, subservient to men, to uphold family honor, and not approach actors outside the community (Ne'eman Haviv, 2020, 2021a,b). The fact that Israeli Arab women are part of a minority group, which has conflicts with the legal authorities, reduces their trust in the authorities and the chance that Arab women will approach either internal or external sources of help.

As noted, Arab society is in the process of transitioning from traditionalism to modernity. The change includes urbanization, structural changes in participation in the labor market and work patterns (especially in the case of women), a rise in educational levels, exposure to media, and detachment from the extended family and traditional social institutions (Elias et al., 2019; Vitman-Schorr and Ayalon, 2020; Lo, 2023). Our findings indicate that there is a negative correlation between the age of the Arab women and their willingness to ask for help, with younger women being more likely to seek sources of support. The change also reflects a willingness on the part of the women to extricate themselves from the cultural trap in which they are.

The women in both groups tend not to turn to their parents for help, especially not to their mothers. Possibly, they do not perceive the mothers as having sufficient social power to help them. But there are differences between the groups in whom they approach for help. As noted, 62.7% of the Jewish women stated that they would turn to a friend for help, whereas only 32.3% of the Arab women indicated that they would turn to a friend. Jewish women were also much more likely to seek help from non-first-degree relatives (24.1% compared to 12.9% of Arab women), from a physician or nurse (37.3 vs. 28.9%), and from a social organization dealing with IPV (54.3 vs. 36.5%).

By contrast, Arab women indicated that they would approach two sources of help more than Jewish women would: social workers (41.7 vs. 37.3%) and religious leaders (27.8 vs. 17.4%). This finding indicates the tendency of Arab women to turn to formal authorities. Over the past decade, there has been a notable rise in awareness among the Arab public in Israel regarding the urgency to establish programs aimed at preventing domestic violence. Consequently, numerous organizations have emerged within Arab society, providing essential support services for women. In many instances, social workers within Arab communities are Arab women themselves, which not only enhances accessibility but also helps to alleviate mistrust.

The tendency of Arab women to seek help from external parties rather than their relatives attests to the sway of traditional culture, which silences women victims. Cuesta-García and Crespo (2022) reviewed numerous studies showing that family reactions and women's fear about what their family members and friends might think acted as a barrier to help-seeking. For example, Abu-Ras (2007) found that Arab immigrant women in the US showed fear of disapproval and of being stigmatized by their families for their decision to seek help.

Shechtman et al. (2018) explained the reluctance to seek help from parties in the community both by the social stigma and the self-stigma associated with it. Public stigma refers to the negative stereotypes that individuals perceive that their society maintains about those who seek help. In previous studies, public stigma has been associated with decreased help-seeking attitudes and intentions (Vogel et al., 2017; Hulley et al., 2023). Individuals internalize these stereotypes and attach negative attitudes to their self-concept, a phenomenon known as self-stigma, which includes feelings of shame, fear of losing self-respect, and isolation (Liang et al., 2005; Shechtman et al., 2018). This explanation clarifies why the Arab women in the present study are less likely to ask for help, underestimated the ability of their mothers to provide support. The choice to turn to official bodies provides women with protection and gives them the strength to face the social pressures in their community.

### Limitations

The main limitation of the study lies in the fact that the women were not directly asked whether they had been victims of IPV. The purpose of the study was to learn about the cultural and social differences in the help-seeking tendency between Jewish and Arab women. Therefore, the study addressed the general population. But, it is possible, that some of the women who participated in the study, were IPV victims, creating a bias in their answers. Moreover, the study examined the women's perception of a theoretical case of IPV: “If you were a victim, whom would you turn to?” Therefore, our results indicate an expectation of behavior rather than actual behavior.

### External validity and generalizability

The study examines differences between two population groups: Jewish and Arab women. Two cultural groups differ from each other: Jewish society is more modern, Western and liberal, whereas Arab society is more conservative, traditional, collectivist and patriarchal. Although the differences between the groups are not necessarily dichotomous, but rather on a continuum, this study can be broadly generalized. The research findings may be relevant to many societies that are in the process of modernization and change, such as traditional immigrant communities living in Western countries.

### Future research

A follow-up study should be conducted among women who identify themselves as victims of IPV, to examine whether they sought help and from which sources. In addition, it is important checking what influenced the decision to contact or not to contact a certain source.

### Prevention and policy implications

Both state agencies and women's organizations in Israel have made attempts to raise awareness of IPV and encourage women to seek help in the case of victimization. It appears, however, that cultural barriers significantly reduce the willingness to seek help. This study shows that it is necessary to act with cultural sensitivity and adapt help options to the culture to which the women belong. Furthermore, the understanding of the barriers facing Arab women, which result in low rates of help seeking within their community, suggests the need to develop intervention programs, that raise the community awareness of the difficulty of escaping the cycle of violence, and offer an enabling place for receiving support. To create a fundamental change, it is necessary to start with educational programs from a young age in schools, with the aim of encouraging solidarity against violence.

## Data availability statement

The original contributions presented in the study are included in the article/supplementary material, further inquiries can be directed to the corresponding authors.

## Ethics statement

The study involving human participants was approved by the Ariel University Ethics Committee (Approval no. AU-criminology-CRI033-APR-2021). Written informed consent to participate in this study was not required from the participants in accordance with the national legislation and the institutional requirements.

## Author contributions

All authors listed have made a substantial, direct, and intellectual contribution to the work and approved it for publication.

## References

[B1] Aboulhassan S. Brumley K. M. (2019). Carrying the burden of a culture: bargaining with patriarchy and the gendered reputation of Arab American women. J. Fam. Issues 40, 637–661. 10.1177/0192513X18821403

[B2] Abu-Ras W. (2007). Cultural beliefs and service utilization by battered Arab immigrant women. Viol. Against Women. 13, 1002–1028. 10.1177/1077801207306019 17898238

[B3] Benita R. (2017). Murder and Attempted Murder of Women With Emphasis on Domestic Violence. Research and Information Center. Jerusalem: Knesset of Israel.

[B4] Ben-Porat A. Levy D. Kattoura O. Dekel R. Itzhaky H. (2021). Domestic violence in Arab society: a comparison of Arab and Jewish women in shelters in Israel. J. Interperson. Viol. 36, NP26-NP45. 10.1177/0886260517731789 29294921

[B5] Central Bureau of Statistics. (2020). The population of Israel at the beginning of 2020. Israel.

[B6] Cheng T. C. Lo C. C. (2015). Racial disparities in intimate partner violence and in seeking help with mental health. J. Interperson. Viol. 30, 3283–3307. 10.1177/0886260514555011 25349016

[B7] Cuesta-García A. Crespo M. (2022). Barriers for help-seeking in female immigrant survivors of intimate partner violence: a systematic review. J. Victimol. 22, 33–59.

[B8] Daoud N. Sergienko R. Shoham-Vardi I. (2020). Intimate partner violence prevalence, recurrence, types, and risk factors among Arab, and Jewish immigrant and nonimmigrant women of childbearing age in Israel. J. Interperson. Viol. 35, 2869–2896. 10.1177/0886260517705665 29294734

[B9] Elias H. Alnabilsy R. Pagorek-Eshel S. (2019). Barriers to receiving support among young Arab women in Israel who have been abused in childhood. Br. J. Soc. Work 49, 2073–2091. 10.1093/bjsw/bcz007

[B10] Faul F. Erdfelder E. Lang A. G. Buchner A. (2007). G^*^ Power 3: a flexible statistical power analysis program for the social, behavioral, and biomedical sciences. Behav. Res. Methods 39, 175–191. 10.3758/BF03193146 17695343

[B11] Frías S. M. Carolina Agoff M. (2015). Between support and vulnerability: examining family support among women victims of intimate partner violence in Mexico. J. Fam. Viol. 30, 277–291. 10.1007/s10896-015-9677-y

[B12] Gilbar O. Charak R. Trujillo O. Cantu J. I. Cavazos V. Lavi I. (2022). Meta-analysis of cyber intimate partner violence perpetration and victimization: different types and their associations with face-to-tace IPV among men and women. Trauma Viol. Abuse 15, 248380221082087. 10.1177/15248380221082087 35603442

[B13] Hulley J. Bailey L. Kirkman G. Gibbs G. R. Gomersall T. Latif A. Jones A. (2023). Intimate partner violence and barriers to help-seeking among Black, Asian, minority ethnic and immigrant women: a qualitative metasynthesis of global research. Trauma Viol. Abuse 24, 1001–1015. 10.1177/15248380211050590 35107333 PMC10012394

[B14] Kim E. Hogge I. (2015). Intimate partner violence among Asian Indian women in the United States: Recognition of abuse and help-seeking attitudes. Int. J. Ment. Health 44, 200–214. 10.1080/00207411.2015.1035073

[B15] Lamers S. Westerhof G. J. Bohlmeijer E. T. ten Klooster P. M. Keyes C. L. (2011). Evaluating the psychometric properties of the mental health continuum-short form (MHC- SF). J. Clinic. Psychol. 67, 99–110. 10.1002/jclp.20741 20973032

[B16] Lelaurain S. Graziani P. Lo Monaco G. (2017). Intimate partner violence and help-seeking: a systematic review and social psychological tracks for future research. Euro. Psychol. 22, 263–281. 10.1027/1016-9040/a000304

[B17] Liang B. Goodman L. Pratyusha T. Weintraub S. (2005). A theoretical framework for understanding help-seeking processes among survivors of intimate partner violence. Am. J. Commun. Psychol. 36, 6. 10.1007/s10464-005-6233-6 16134045

[B18] Lo I. P. Y. (2023). Violence in the “double closet”: female same-sex intimate partner violence and minority stress in China. J. Lesbian Stud. 27, 137–145. 10.1080/10894160.2022.2091732 35757991

[B19] MacGregor J. C. Oliver C. L. MacQuarrie B. J. Wathen C. N. (2021). Intimate partner violence and work: a scoping review of published research. Trauma Viol. Abuse 22, 717–727. 10.1177/1524838019881746 31615345

[B20] Ne'eman Haviv V. (2020). Personal elements in culture-based violence? The case of honor killing-a brief report. J. Forensic Psychiatr. Psychol. 31, 807–813. 10.1080/14789949.2020.1786595

[B21] Ne'eman-Haviv V. (2021a). Israeli Arabs' acculturation patterns and attitudes toward honor killings. Int. J. Intercult. Relat. 85, 104–111. 10.1016/j.ijintrel.2021.09.006

[B22] Ne'eman-Haviv V. (2021b). Attitudes of Arab Israeli students towards honour killings. J. Gender Stud. 30, 18–28. 10.1080/09589236.2020.1768831

[B23] Padilla-Medina D. M. Small E. Pavlova Nikolova S. (2023). Exploring help-seeking predictors among Colombian victims of intimate partner violence in different age groups. Viol. Again. Women 29, 202–228. 10.1177/10778012221088308 35791515

[B24] Potter L. C. Morris R. Hegarty K. García-Moreno C. Feder G. (2021). Categories and health impacts of intimate partner violence in the World Health Organization multi-country study on women's health and domestic violence. Int. J. Epidemiol. 50, 652–662. 10.1093/ije/dyaa220 33326019

[B25] Robinson S. R. Ravi K. Voth Schrag R. J. (2021). A systematic review of barriers to formal help seeking for adult survivors of IPV in the United States, 2005–2019. Trauma Viol. Abuse 22, 1279–1295. 10.1177/1524838020916254 32266870

[B26] Selestine V. Harvey S. Mshana G. Kapiga S. andn Lees S. (2023). The role of structural factors in support-seeking among women experiencing intimate partner violence (IPV) in Mwanza, Tanzania: findings from a qualitative study. Viol. Again. Women 29, 1024–1043. 10.1177/10778012221077130 35213259

[B27] Shechtman Z. Alim E. Brenner R. E. Vogel D. L. (2018). Public stigma, self- stigma, and group therapy help-seeking intentions among clinical and non-clinical Arab adults in Israel. Int. J. Cult. Ment. Health 11, 595–604. 10.1080/17542863.2018.1461913

[B28] Shrira A. Bodner E. Palgi Y. (2016). Positivity ratio of flourishing individuals: examining the moderation effects of methodological variations and chronological age. J. Positiv. Psychol. 11, 109–123. 10.1080/17439760.2015.1037857

[B29] Shwartz N. O'Rourke N. Daoud N. (2022). Pathways linking intimate partner violence and postpartum depression among Jewish and Arab women in Israel. J. Interperson. Viol. 37, 301–321. 10.1177/0886260520908022 32167400

[B30] Spencer C. Mallory A. B. Cafferky B. M. Kimmes J. G. Beck A. R. Stith S. M. (2019). Mental health factors and intimate partner violence perpetration and victimization: a meta-analysis. Psychol. Viol. 9, 1–17. 10.1037/vio0000156

[B31] Stark E. Hester M. (2019). Coercive control: update and review. Viol. Women 25, 81–104. 10.1177/1077801218816191 30803427

[B32] Sweet P. L. (2019). The sociology of gaslighting. Am. Sociologic. Rev. 84, 851–875. 10.1177/0003122419874843

[B33] Tengku Hassan T. N. F. Ali S. H. Salleh H. (2015). Patterns of help-seeking among women experiencing intimate partner violence in Malaysia. Asian J. Women's Stud. 21, 77–92. 10.1080/12259276.2015.1029226

[B34] Turner R. J. Brown R. L. (2010). Social support and mental health. A handbook for the study of mental health: social contexts, theories, and systems. 2, 200–212. 10.1017/CBO9780511984945.014

[B35] van Berkel H. K. (2009). The Relationship Between Personality, Coping Styles and Stress, Anxiety and Depression. Christchurch: University of Canterbury.

[B36] Vitman-Schorr A. Ayalon L. (2020). The changing status of Israeli Arab women as reflected in their role as main caregivers. J. Fam. Issues 41, 2203–2222. 10.1177/0192513X19898829

[B37] Vogel D. L. Strass H. A. Heath P. J. Al-Darmaki F. R. Armstrong P. I. Baptista M. N. Zlati A. (2017). Stigma of seeking psychological services: examining college students across ten countries/regions. Counsel. Psychol. 45, 170–192. 10.1177/0011000016671411

[B38] Waller B. Y. Joyce P. A. Quinn C. R. Hassan Shaari A. A. Boyd D. T. (2023). “I am the one that needs help”: the theory of help-seeking behavior for survivors of intimate partner violence. J. Interperson. Viol. 38, 288–310. 10.1177/08862605221084340 35350920 PMC9519802

